# Beyond Donor-Specific Antibodies: The Unseen Role of Non-Donor-Specific Anti-human Leukocyte Antigen (HLA) Antibodies in Rejection

**DOI:** 10.7759/cureus.104798

**Published:** 2026-03-06

**Authors:** Pranjal Kashiv, Manish Balwani, Amit S Pasari, Vivek Kute

**Affiliations:** 1 Nephrology, All India Institute of Medical Sciences, Nagpur, Nagpur, IND; 2 Nephrology, Saraswati Kidney Care Center, Nagpur, IND; 3 Nephrology, Jawaharlal Nehru Medical College, Wardha, IND; 4 Nephrology, Institute of Kidney Diseases and Research Centre, Ahmedabad, IND

**Keywords:** allograft biopsy, antibody-mediated rejection, kidney transplantation, microvascular inflammation, microvascular injury, non–donor-specific anti-hla antibodies, single antigen bead assay

## Abstract

Antibody-mediated rejection (ABMR) is conventionally defined by the presence of donor-specific anti-human leukocyte antigen (HLA) antibodies (DSA), characteristic microvascular injury on allograft biopsy, and evidence of complement activation, most commonly reflected by C4d deposition. We report the case of a 46-year-old woman who underwent a fully HLA-matched live-related kidney transplant with negative crossmatches and no detectable DSA, but with high-mean fluorescence intensity (MFI) non-DSA (NDSA). Sixteen months post-transplant, she developed unexplained graft dysfunction. Allograft biopsy demonstrated moderate-to-severe interstitial inflammation and tubulitis (Banff Grade IA) with associated microvascular inflammation characterized by glomerulitis and peritubular capillaritis, and C4d negativity. In the context of prior high-MFI NDSA and suspicious antibody-mediated features, the patient received combined immune-directed therapy, including corticosteroids, plasmapheresis, intravenous immunoglobulin, rituximab, and optimization of maintenance immunosuppression, resulting in significant improvement in graft function. She remained under continuous follow-up for six months post treatment, with stable graft function, normal serum creatinine levels, and no recurrence of rejection. This case highlights a seronegative ABMR-like phenotype in which NDSA may contribute to graft injury despite negative DSA and C4d, underscoring the limitations of a strictly DSA-centric diagnostic framework.

## Introduction

Antibody-mediated rejection (ABMR) is traditionally defined by the presence of donor-specific anti-human leukocyte antigen (HLA) antibodies (DSA), characteristic microvascular injury on allograft biopsy, and evidence of complement activation, most commonly reflected by C4d deposition [1]. Contemporary diagnostic frameworks place substantial emphasis on circulating DSA as a central criterion for diagnosing ABMR and guiding therapeutic decision-making [2]. However, an increasing subset of kidney transplant recipients present with histological features strongly suggestive of antibody-mediated injury despite the absence of detectable DSA or C4d positivity, creating significant diagnostic and therapeutic uncertainty [3].

Non-DSA (NDSA), identified as high mean fluorescence intensity (MFI) reactivity on single-antigen bead (SAB) assays without specificity to donor HLA alleles, are increasingly recognised as potentially pathogenic rather than incidental markers of sensitization [4]. SAB assays are Luminex-based solid-phase immunoassays that detect anti-HLA antibodies against individual HLA antigens, with MFI serving as a semi-quantitative indicator of antibody strength. These antibodies may contribute to endothelial injury through recognition of shared or cross-reactive epitopes, eplet-level mismatches, or complement-independent immune mechanisms, particularly in previously sensitised individuals [5,6]. Complement-binding capacity and antibody characteristics, rather than donor specificity alone, have also been shown to influence graft outcomes, further supporting antibody-mediated injury beyond classical DSA paradigms [7].

Emerging clinical studies have demonstrated that grafts exposed to NDSA or pre-existing anti-HLA antibodies can exhibit microvascular inflammation and adverse outcomes comparable to classical ABMR, even in the absence of detectable DSA [3,8]. Despite these observations, standardized diagnostic criteria and management strategies for such seronegative rejection phenotypes remain lacking [2,9,10].

We report a case of biopsy-proven rejection with microvascular inflammation in the absence of detectable DSA. Despite increasing recognition of DSA-negative phenotypes, the clinical significance of NDSA and optimal management strategies remains uncertain. Documenting such cases with careful clinicopathologic correlation is essential to refining diagnostic frameworks and informing therapeutic decision-making beyond a strictly DSA-centric paradigm.

## Case presentation

A 46-year-old woman with end-stage kidney disease on maintenance hemodialysis for 56 months, secondary to long-standing hypertension with suspected chronic glomerulonephritis, was evaluated for kidney transplantation. She was dialyzing via a well-functioning left radio-cephalic arteriovenous fistula.

The patient had multiple historical risk factors for alloimmune sensitization. She had received several blood transfusions during the course of dialysis. In February 2021, she had a documented COVID-19 infection that required treatment with remdesivir and systemic corticosteroids. She also had a history of two full-term pregnancies. There was no prior history of organ transplantation.

Initial transplant planning involved her mother as a prospective living donor. However, complement-dependent cytotoxicity (CDC) crossmatch testing was positive, indicating significant preformed anti-donor reactivity, and transplantation with the mother was not pursued further. Subsequently, her brother was evaluated as an alternative living donor. The donor and recipient were ABO compatible.

A comprehensive immunological workup was undertaken. High-resolution HLA typing was performed using intermediate-resolution sequence-specific oligonucleotide (SSO) methodology on the Luminex platform. Typing included HLA-A, HLA-B, HLA-C, HLA-DRB1, HLA-DQA1, HLA-DQB1, HLA-DPA1, and HLA-DPB1 loci. The donor and recipient were found to be fully matched at all tested loci, confirming a complete 12/12 HLA match.

CDC crossmatch performed using donor lymphocytes showed less than 10% cell lysis and was reported as negative. Flow cytometry crossmatch for both T and B lymphocytes was also negative, with no significant fluorescence shift observed in either population. Autologous crossmatch controls were within reference ranges.

**Figure 1:**
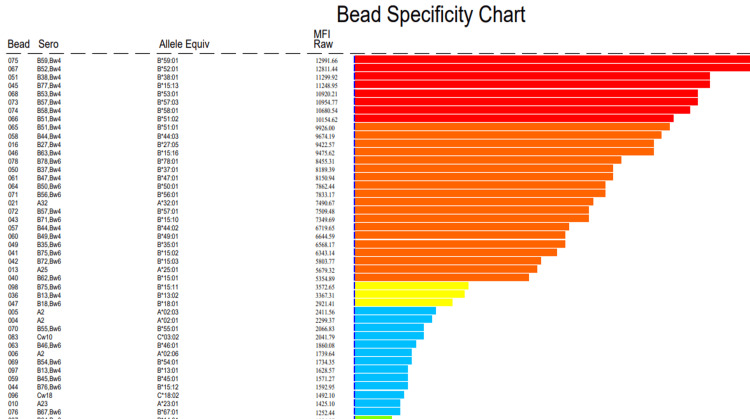
SAD assay demonstrating high-MFI, non–donor-specific anti-HLA Class I antibody reactivity Single antigen bead (SAB) assay showing anti-HLA Class I antibody reactivity with multiple beads exhibiting high mean fluorescence intensity (MFI) values. Several HLA Class I specificities demonstrate MFI values exceeding commonly used pathogenic thresholds (>5,000–10,000), indicating a substantial antibody burden. None of the detected antibody specificities corresponded to the donor’s HLA alleles, consistent with non–donor-specific anti-HLA antibody (NDSA) reactivity. HLA: human leukocyte antigen

**Figure 2:**
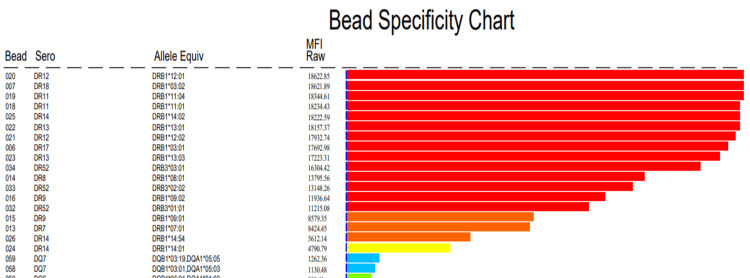
SAB assay demonstrating high-MFI non-donor-specific anti-HLA Class II antibody reactivity Single antigen bead (SAB) assay showing anti-HLA Class II antibody reactivity with multiple DR and DQ specificities exhibiting high mean fluorescence intensity (MFI) values, several exceeding commonly accepted pathogenic thresholds (>5,000–10,000). None of the detected antibody specificities corresponded to donor HLA Class II alleles, consistent with non–donor-specific anti-HLA antibody (NDSA) reactivity. HLA: human leukocyte antigen

Panel reactive antibody (PRA) screening using Luminex bead-based assays demonstrated positivity for both HLA Class I and Class II antibodies. Testing for non-HLA antibodies, including MICA antibodies, was negative. In view of PRA positivity, SAB assays were performed for detailed antibody specificity assessment, and revealed multiple high-MFI antibodies against HLA Class I antigens, with peak MFI values reaching up to approximately 13,000, and against HLA Class II antigens, with peak values up to approximately 18,600. Importantly, none of the detected antibody specificities corresponded to the donor’s HLA alleles. Final DSA assessment using SAB for both Class I and Class II was negative (Figure 1, 2).

Based on the absence of detectable DSA, negative CDC and flow crossmatches, and a complete HLA match, the patient proceeded to transplantation. She underwent a live-related renal transplant with her 50-year-old brother as the donor. The immediate postoperative period was uncomplicated.

Induction therapy was not administered as per institutional protocol. Maintenance immunosuppression consisted of tacrolimus, mycophenolate mofetil, and corticosteroids. The patient received intravenous (IV) methylprednisolone 500 mg daily for three consecutive days. Tacrolimus was initiated at 0.1 mg/kg/day in two divided doses, mycophenolate mofetil at 2,000 mg/day in two divided doses, and oral prednisolone was started on postoperative day four at 0.5 mg/kg/day.

One month later, the patient developed post-transplant diabetes mellitus, attributed to tacrolimus. Tacrolimus was therefore discontinued and replaced with cyclosporine. Later the same month, she developed gastrointestinal intolerance manifesting as diarrhea, suspected to be related to mycophenolate mofetil. The dose was initially reduced and subsequently switched to mycophenolate sodium. Cyclosporine levels were monitored using C2 assays and remained within therapeutic range. However, three months after this, cyclosporine was discontinued due to cosmetically distressing facial hypertrichosis, and sirolimus was introduced. Therapeutic drug monitoring showed stable sirolimus levels following dose adjustments.

One year and one month after the transplantation, the patient developed progressive bilateral visual impairment. Ophthalmological evaluation led to a diagnosis of ischemic optic atrophy. In the absence of alternative etiologies, sirolimus-related toxicity was suspected, and sirolimus was discontinued. Cyclosporine was reintroduced at a reduced dose with subsequent titration based on drug levels.

Graft function remained stable through this period, with serum creatinine consistently between 0.94 and 0.97 mg/dL. After another three months, a gradual rise in serum creatinine was noted, increasing to 2.13 mg/dL and further to 2.56 mg/dL in 10 days. The patient was asymptomatic; urine analysis and culture were unremarkable, cytomegalovirus and BK virus screening were negative, and imaging excluded obstruction or vascular compromise. Drug levels were within therapeutic range.

**Figure 3:**
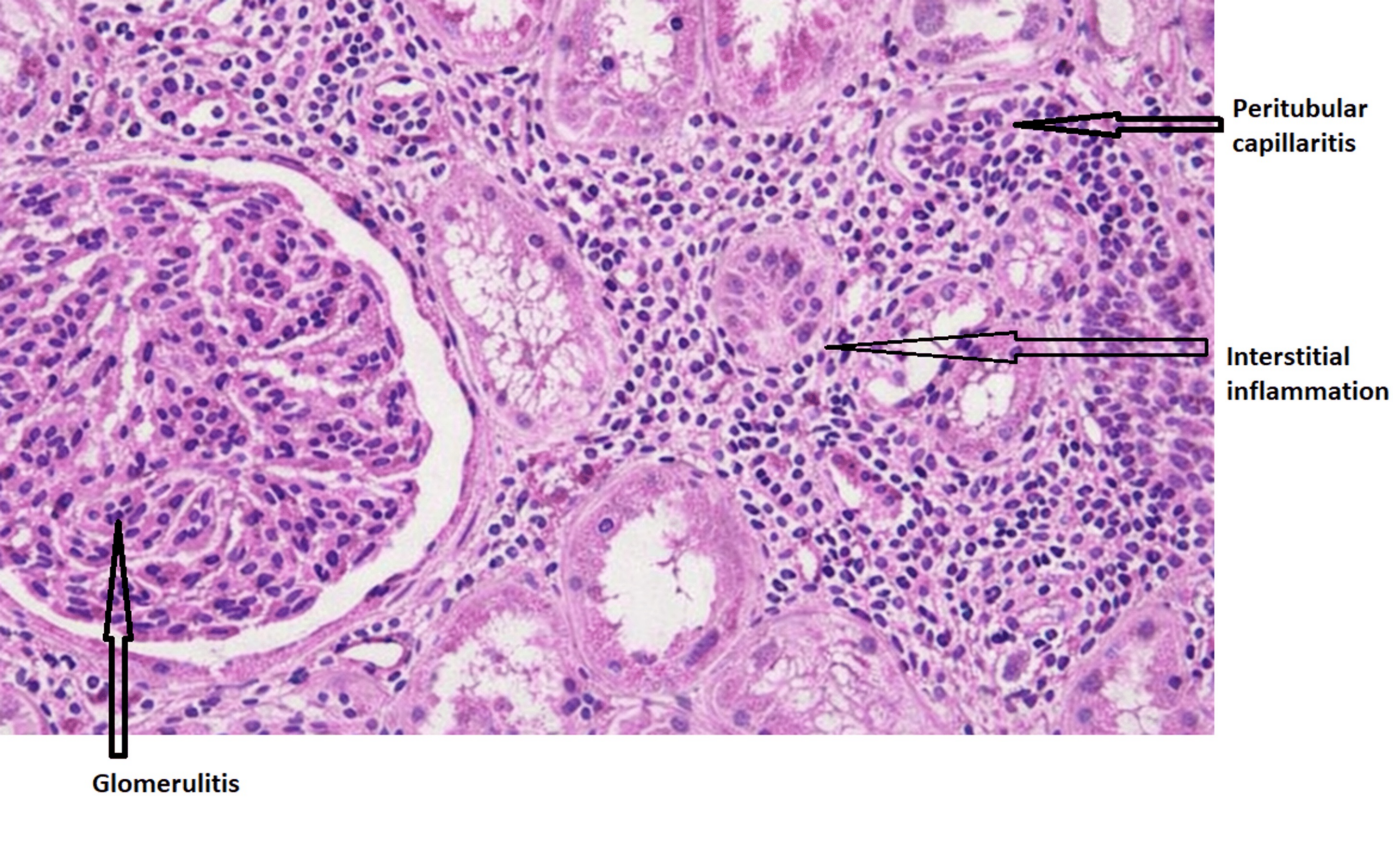
Renal allograft biopsy showing interstitial inflammation with glomerulitis and focal peritubular capillaritis. Hematoxylin and eosin–stained section of a renal allograft biopsy at ×200 magnification demonstrating moderate–severe interstitial mononuclear inflammation with glomerulitis and focal peritubular capillaritis.

**Figure 4:**
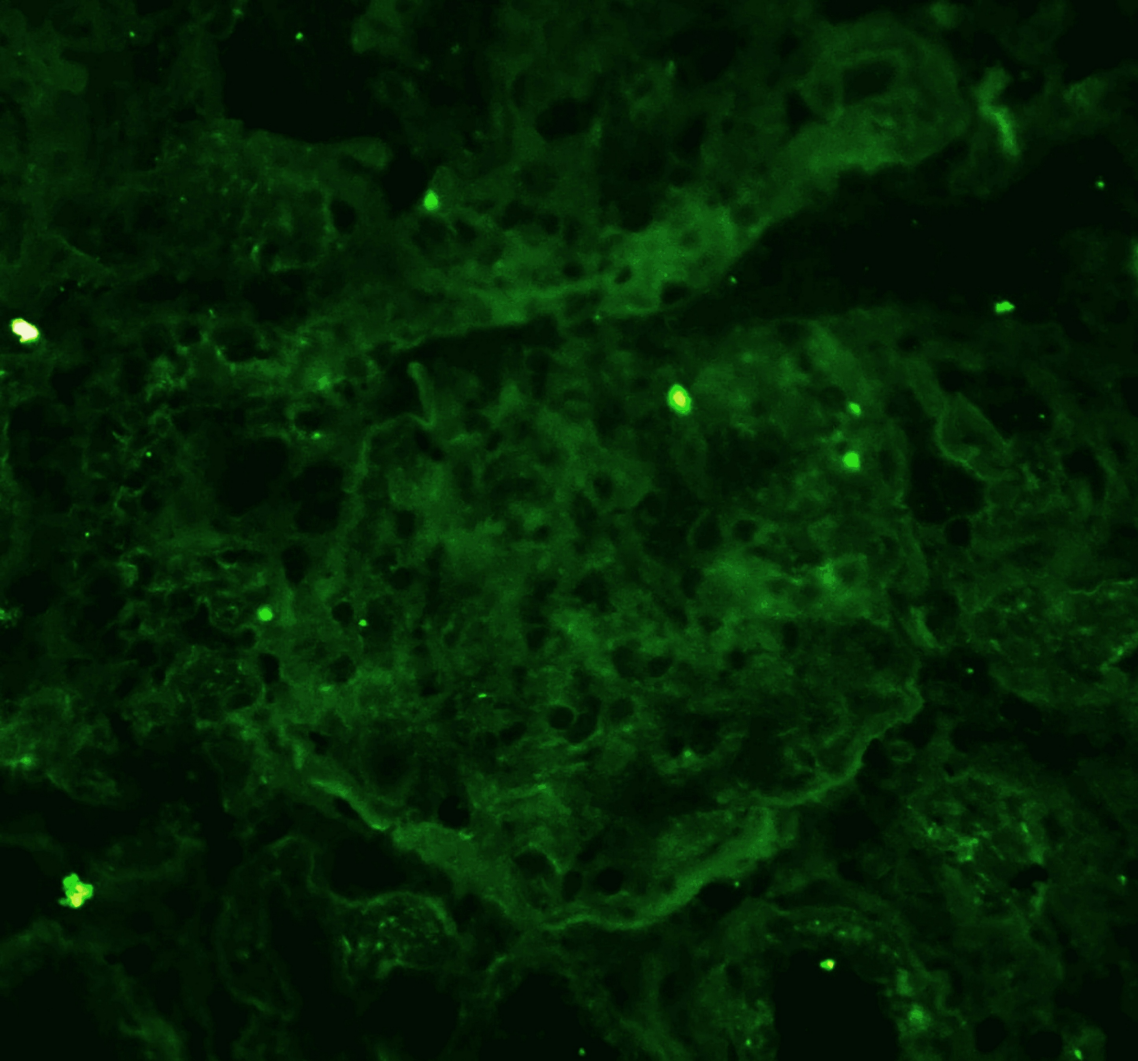
Immunofluorescence study of renal allograft biopsy. Immunofluorescence microscopy of renal allograft biopsy at ×200 magnification showing no specific immune complex deposition.

Given the unexplained deterioration in graft function, a renal allograft biopsy was performed. Histopathological examination, interpreted according to Banff 2022 criteria, revealed moderate-to-severe interstitial inflammation and tubulitis (Banff Grade IA) with associated microvascular inflammation characterized by glomerulitis and peritubular capillaritis [11]. Chronic changes were mild. C4d immunohistochemistry was negative, and there was no evidence of transplant glomerulopathy or vascular intimal arteritis (Figures 3, 4).

The biopsy was interpreted as T cell-mediated rejection (Banff Grade IA) with features suspicious for concomitant antibody-mediated rejection. Repeat DSA testing could not be performed due to financial constraints. In view of the histological findings and prior demonstration of high-MFI non-donor-specific anti-HLA antibodies, empiric treatment for antibody-mediated injury was initiated.

The patient received pulse methylprednisolone, followed by plasma exchange and IV immunoglobulin (IVIG) therapy. Rituximab was administered for B-cell depletion, and immunosuppression was intensified with reintroduction of tacrolimus. Following treatment, serum creatinine showed a progressive decline, reaching 1.04 mg/dL by one month, consistent with reversal of rejection.

The patient was subsequently maintained on optimized maintenance immunosuppression and continued under regular outpatient follow-up. Over the ensuing six months post treatment, graft function remained stable, with serum creatinine levels ranging between 0.6 and 1.0 mg/dL. At the most recent follow-up in January 2026, one year after developing the developed progressive bilateral visual impairment, serum creatinine was 0.9 mg/dL. There has been no clinical or biochemical evidence of recurrent rejection, and the patient remains clinically stable.

## Discussion

This case illustrates a clinically and mechanistically important form of graft injury that challenges the conventional donor-specific antibody-centric framework of antibody-mediated rejection [1]. Despite a complete 12/12 HLA match, negative CDC and flow crossmatches, absence of detectable DSA, negative MICA antibodies, and C4d negativity, the patient developed biopsy-proven rejection with significant microvascular inflammation and demonstrated a robust response to antibody-directed therapy [2]. The only immunologic abnormality consistently present was a burden of high-MFI NDSA, suggesting a pathogenic role for these antibodies in driving graft injury [3,4].

NDSA as a pathogenic entity beyond “bystander” antibodies

Historically, NDSA were considered epiphenomena-markers of sensitization without direct pathogenic relevance [1]. However, accumulating evidence over the past decade has fundamentally challenged this assumption. Senev et al. demonstrated that kidney transplant recipients with microvascular inflammation and negative DSA had graft survival comparable to classical ABMR, particularly when antibody burden and prior sensitization were present [3]. Importantly, these patients frequently exhibited high-MFI anti-HLA reactivity without donor specificity, similar to the immunologic profile observed in our patient.

Severova-Andreevska and colleagues further reported that patients with isolated NDSA, in the absence of detectable DSA, could develop significant glomerulitis and peritubular capillaritis and showed clinical improvement following plasmapheresis and IVIG [4]. These observations support the concept that NDSA are not merely serologic noise but may participate directly in endothelial injury.

Our case aligns closely with these findings. The presence of multiple Class I and II anti-HLA antibodies with MFIs exceeding commonly accepted pathogenic thresholds (>5,000-10,000), coupled with biopsy-proven microvascular inflammation, suggests that antibody burden and affinity, rather than donor specificity alone, may determine pathogenic potential in certain immunologic contexts [5,6].

Mechanistic considerations: how NDSA can cause injury in a fully matched transplant

Several biologically plausible mechanisms may explain antibody-mediated injury in the absence of donor specificity. Anti-HLA antibodies may recognize shared or cross-reactive epitopes across HLA alleles, including public epitopes or structurally conserved eplets not captured by conventional allele-level matching [10]. Eplet mismatch analysis has demonstrated that antibodies directed against shared eplets can bind donor endothelium despite apparent HLA identity, particularly when assessed using intermediate-resolution typing [5].

In addition, non-complement-fixing antibodies may exert pathogenic effects through Fc-mediated activation of endothelial cells, recruitment of innate immune cells, or induction of pro-inflammatory gene expression without C4d deposition [7]. This may explain the increasingly recognised phenotype of C4d-negative ABMR with prominent microvascular inflammation, as observed in this case [2,3].

The patient’s extensive sensitization history, including multiple transfusions, prior pregnancies, and viral immune activation during COVID-19 infection, likely contributed to a primed humoral immune repertoire [6,8]. Such pre-existing memory responses may become clinically relevant when immunosuppression is modified, as occurred repeatedly in this patient due to metabolic, gastrointestinal, and drug-related toxicities.

Histology over serology: diagnostic implications

This case reinforces a critical principle increasingly emphasised in contemporary transplant pathology: histology should not be subordinated to serology [2]. The Banff 2022 update explicitly acknowledges that antibody-mediated mechanisms may operate in the absence of detectable DSA and that microvascular inflammation retains diagnostic and prognostic significance even when serologic criteria are unmet [2].

Our patient’s biopsy demonstrated a mixed rejection phenotype, with both T cell-mediated rejection and features suspicious for ABMR. Such overlap is well described in sensitized recipients and underscores the limitations of binary diagnostic categories [3,9]. Importantly, the clinical response to antibody-targeted therapy provides functional validation of the pathogenic relevance of humoral mechanisms in this case [4].

Therapeutic and conceptual implications

There is no standardized treatment strategy for suspected NDSA-mediated rejection. However, available evidence, including the present case, suggests that empiric immune-directed therapy may be justified when biopsy findings are compelling and alternative explanations are excluded [3,4]. In our patient, the biopsy demonstrated Banff Grade IA T cell-mediated rejection with features suspicious for concomitant antibody-mediated rejection, and treatment was therefore directed at a mixed rejection phenotype. The marked improvement in graft function following combined therapy, including plasmapheresis, IVIG, rituximab, and optimization of calcineurin-based immunosuppression, is consistent with response in a mixed immune-mediated process and supports a clinically relevant humoral contribution in this DSA-undetected setting. Although repeat DSA testing was not feasible in our case due to financial constraints, de novo DSA cannot be definitively excluded.

More broadly, this case contributes to a growing body of literature questioning the adequacy of a purely DSA-centric model of rejection. It highlights the need for refined immunologic risk stratification incorporating antibody burden, epitope analysis, and immune history, rather than reliance on donor specificity alone.

## Conclusions

This case demonstrates that clinically significant rejection can occur in the complete absence of detectable donor-specific antibodies, even in a fully HLA-matched kidney transplant. High-MFI NDSA, in the appropriate clinical and histological context, may act as pathogenic drivers of graft injury and produce a phenotype indistinguishable from classical antibody-mediated rejection. The presence of microvascular inflammation on biopsy should prompt consideration of humoral mechanisms regardless of serologic DSA status. Recognition of this entity is critical, as timely antibody-directed therapy may result in meaningful graft recovery. These observations underscore the limitations of a strictly DSA-centric framework and highlight the need for integrated interpretation of immunologic history, antibody burden, and histopathology in the evaluation of unexplained allograft dysfunction.
